# On Asthma: Its Pathology and Treatment

**Published:** 1864-03

**Authors:** 


ON ASTHMA: ITS PATHOLOGY AND TREATMENT. By HENRY HYDE SALTER, M.D., F.R.S., Fellow of the Royal College of Physicians; Assistant Physician to the Charing Cross Hospital; Lecturer on Physiology and Pathology at the Charing Cross Hospital Medical School. Philadelphia: BLANCHARD & LEA. 1864.

This is an octavo volume of 260 pages, neatly bound in cloth. The author discusses the nature and treatment of Asthma in a very thorough and interesting manner. His views regarding the pathology of the disease may be inferred from the following propositions:—

"1. That asthma is essentially, and, with perhaps the exception of a single class of cases, exclusively a nervous disease: that the nervous system is the seat of the essential pathological condition.

"2. That the phenomena of asthma—the distressing sensation and the demand for extraordinary respiratory efforts—immediately depend upon a spastic contraction of the fibre-cells of organic or unstriped muscle, which minute anatomy has demonstrated to exist in the bronchial tubes.

"3. That these phenomena are those of excito-motory or reflex action.

"4. That the extent to which the nervous system is involved differs very much in different cases, being in some cases restricted to the nervous system of the air-passages themselves.

"5. That in a large number of cases the pneumogastric nerve, both in its gastric and pulmonary portions, is the seat of the disease.

"6. That there is a large class of cases in which the nervous circuit between the source of irritation and the seat of the resulting mascular phenomena involves other portions of the nervous system besides the pneumogastric.

"7. That there are other cases in which the source of irritation, giving rise to the asthmatic paroxysm, appears to be central—in the brain; consequently, in which the action, though excito-motory, is not reflex.

"8. That there is yet a class of cases in which the exciting cause of the paroxysms appears to be essentially humoral."

These propositions are thoroughly discussed and sustained by an interesting variety of facts and cases. But we have neither time nor space to give even an outline of the treatise before us. It is one of the books that will repay the practitioner for a careful perusal.

For sale by W. B. KEEN & Co., Lake street, Chicago.

